# Comparison of minimal access and open breast surgery: a propensity score-matched study on postoperative immune function in breast cancer

**DOI:** 10.1186/s12957-024-03447-w

**Published:** 2024-07-16

**Authors:** QiHua Jiang, Jing Liao, JunTao Tan, Hai Hu

**Affiliations:** 1 Department of Breast Surgery, Third Hospital of Nanchang, No. 2, Xiangshan South Road, Xi hu District, Nanchang City, Jiangxi Province China; 2 Department of General Surgery, Third Hospital of Nanchang, No. 2, Xiangshan South Road, Xi hu District, Nanchang City, Jiangxi Province China; 3 Jiangxi Province Key Laboratory of Breast Diseases, Third Hospital of Nanchang, No. 1268, Jiuzhou Street, Chaoyang New Town, Xihu District, Nanchang City, Jiangxi Province China

**Keywords:** Minimal access breast surgery, Endoscopic, Immune function, Breast cancer

## Abstract

**Background:**

Minimal access breast surgery (MABS) is commonly employed in the management of breast cancer, but there is limited research on the postoperative immune function associated with MABS.

**Objective:**

This study aimed to assess the postoperative immune function in breast patients who underwent MABS or conventional open breast surgery (COBS).

**Methods:**

We retrospectively analyzed the medical records of 829 breast cancer patients treated with either MABS or COBS at a single hospital between January 2020 and June 2023. Among them, 116 matched pairs were obtained through 1:1 propensity score matching (PSM). Flow cytometry was used to measure the percentages of CD3^+^, CD4^+^, and CD8^+^ cells, as well as the CD4^+^/CD8^+^ ratio, on three different time points: preoperative day 1 (PreD1), postoperative day 1 (PostD1), and postoperative day 7 (PostD7).

**Results:**

Both the MABS and COBS groups demonstrated a significant reduction in the percentages of CD3^+^, CD4^+^, and CD8^+^ cells, along with the CD4^+^/CD8^+^ ratio, from PreD1 to PostD1. Interestingly, the MABS group showed a reversal of these parameters, returning to preoperative levels by PostD7. Conversely, the COBS group showed an increase in these parameters from PostD1 to PostD7, but they still remained significantly lower than preoperative levels at PostD7.

**Conclusion:**

MABS treatment may result in reduced postoperative immune suppression and faster recovery of preoperative immune function compared to COBS in patients.

## Background

According to the latest Global Cancer Statistics report, there were approximately 19.3 million novel occurrences of cancer observed globally, with 9.23 million cases exclusively affecting the female population, further breaking down to 2.26 million incidents of breast cancer in women [1]. Breast cancer has overtaken lung cancer as the primary instigator of cancer and currently ranks as the fifth most prevalent factor contributing to cancer-related fatalities worldwide [2, 3]. The management of breast cancer requires a comprehensive approach that includes surgical interventions as the primary treatment modality, along with additional measures such as radiotherapy, chemotherapy, endocrine therapy, and targeted therapy [4–6].

Minimally invasive breast surgery (MABS), including endoscopic and robotic techniques, has become increasingly prevalent in procedures like nipple sparing mastectomy, lymph node dissection, breast conservation surgery and reconstruction [7–10]. . MABS offers multiple advantages over conventional open breast surgery (COBS), including smaller incisions, reduced trauma, less bleeding, decreased postoperative pain, faster recovery time, and improved cosmetic outcomes [11–14]. However, one challenge in MABS is the absence of a natural cavity in the breast area, requiring the administration of a lipolysis solution to create the necessary operating space. This has the potential to cause harm to the fat and surrounding tissues [15]. Tissue damage can trigger an immune response to prevent further harm, and surgical trauma may lead to a systemic inflammatory response, impacting postoperative immune function [16, 17]. Postoperative immune suppression is notably more severe in open abdomen and thoracic surgeries than in minimally invasive endoscopic procedures [18, 19]. For example, postoperative cellular-mediated immune dysfunction is significantly higher in open colectomy than laparoscopic colectomy. Similar findings have been observed in patients with pulmonary malignancies, where aggressive treatment is linked to postoperative immune suppression. The current remaining issue is whether postoperative immune suppression following MABS is equivalent to or attenuated compared to COBS.

Given the potential for postoperative immune suppression and postoperative complications linked to the open procedure, it is crucial to explore and address this particular question. To evaluate the postoperative immune suppression between MABS and COBS, we conducted a propensity score-matched (PSM) retrospective analysis in breast cancer patients treated with either approach at a single large medical institution.

## Methods

### Study population

Between June 2020 and July 2023, 829 breast cancer patients were included in the database of Third Hospital of Nanchang. Inclusion criteria for the study comprised female patients between the ages of 18 and 75, with cancer stages ranging from 0 to III, and no prior history of surgery. Exclusion criteria comprised of incomplete fundamental information and previous administration of neoadjuvant therapies such as radiotherapy and/or chemotherapy, and patients with immune disorders.

The surgical procedures performed on patients encompassed modified radical mastectomy, subcutaneous adenoectomy, simple-mastectomy, breast conservative surgery, and breast reconstruction with COBS, as well as endoscopic or robotic breast surgery.

**Fig. 1:**
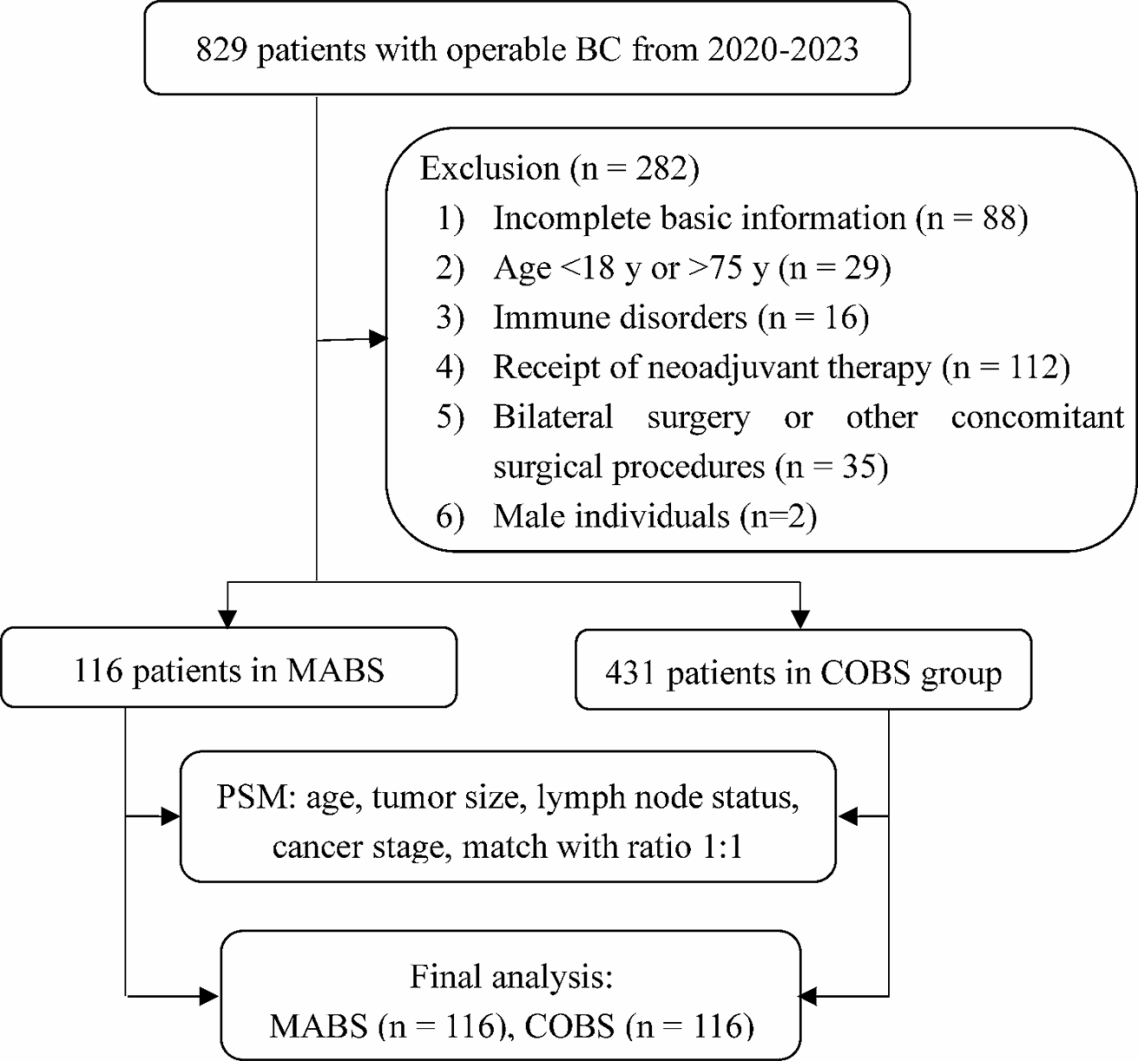
Patient flow diagram. BC, breast cancer; MABS, minimally access breast surgery; COBS, conventional open breast surgery; PSM, propensity score matching

Ultimately, 232 patients were included for analysis as depicted in Fig. 1. Ethical approval for the study was granted by the hospital’s ethics committee in compliance with the Helsinki Declaration.

**Fig. 2:**
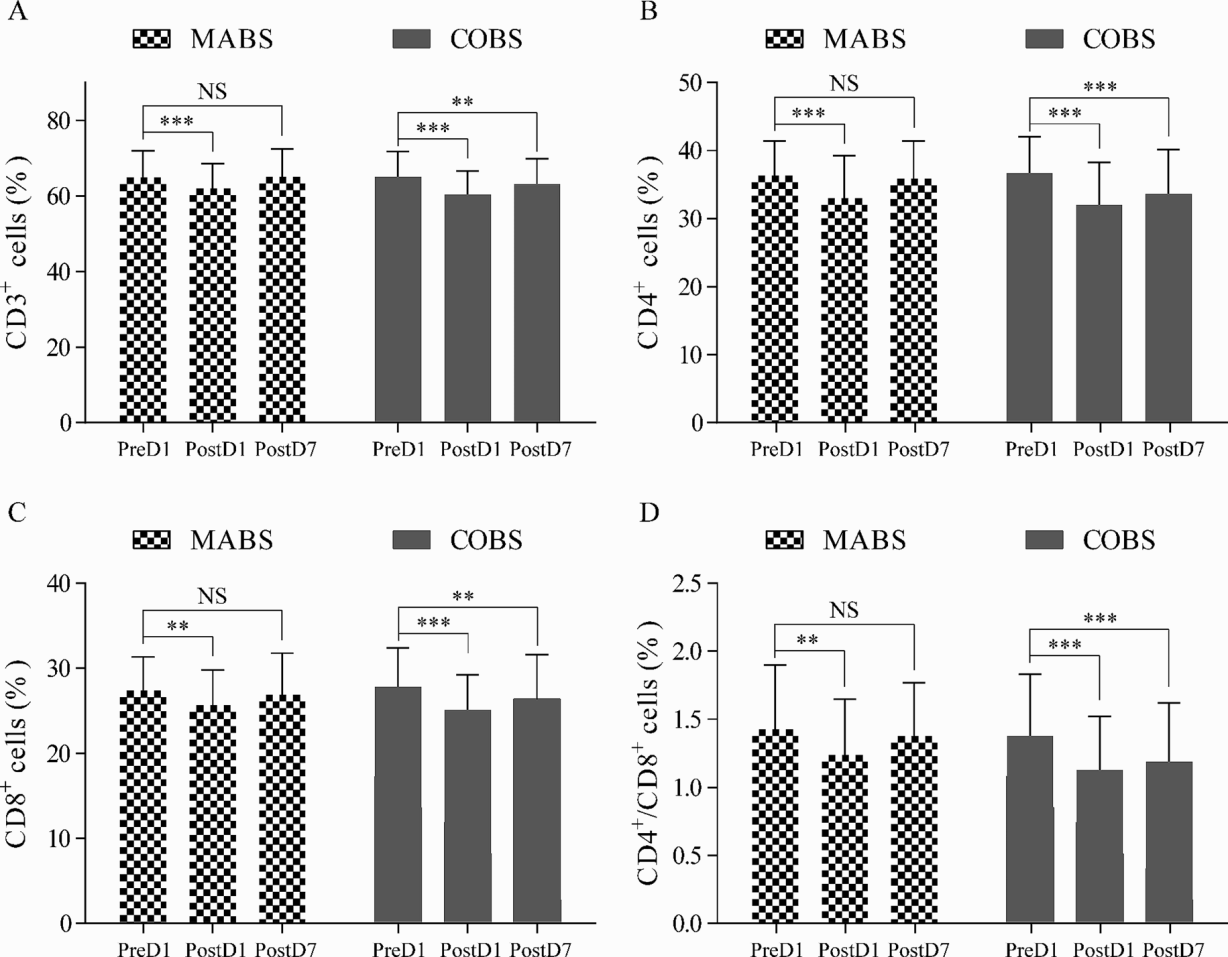
Comparison of proportions of (**A**) CD3^+^ cells, (**B**) CD4^+^ cells, (**C**) CD8^+^ cells, and (**D**) the CD4^+^/CD8^+^ ratio at various time points in patients underwent MABS or COBS. Values are expressed as mean ± SD. MABS, minimally access breast surgery; COBS, conventional open breast surgery; PreD, preoperative day; PostD, postoperative day; ns, no significance; *P < 0.05, **P < 0.01, ***P < 0.001

### Study design

Patients were categorized into two groups: the MABS group, encompassing individuals who experienced endoscopic, endoscopy-aided, and robotic-supported surgeries, and the COBS group, encompassing those who had COBS operations. The primary outcome focused on the evaluation of postoperative immune function, assessed by the percentages of CD3^+^, CD4^+^, and CD8^+^ lymphocytes, along with the CD4^+^/CD8^+^ ratio. Within each treatment group, postoperative immune function was compared at different time intervals, namely preoperative day (PreD) 1 and postoperative days (PostD) 1 and 7. Specifically, the proportion of immune cells in peripheral venous blood was measured by flow cytometry on PreD 1, PostD 1, and PostD 7. Furthermore, a comparison was conducted between the two groups at the same time interval. Additionally, demographic and clinicopathological factors, alongside postoperative morbidity rates, were also compared.

To control for potential confounding factors and ensure comparability between the two surgical groups, a propensity score matching (PSM) approach was utilized. The propensity score was calculated using logistic regression analysis, incorporating covariates such as age, body mass index (BMI), tumor size, cancer stage, and lymph node status, which could influence the choice of surgical procedure and outcomes. This analysis was performed using the MatchIt package in R software (R Foundation). A 1:1 nearest-neighbor matching technique was applied without specifying a caliper value to ensure tight matching. Following the matching process, the balance of covariates was assessed, with all P values for the matched samples exceeding 0.05, indicating a successful balance between the groups. Each group, post-matching, consisted of 116 patients.

### Statistical analysis

Statistical analyses were conducted using SPSS 21.0 for basic statistics, including means ± SD for continuous variables and percentages for categorical data. For propensity score matching (PSM), we employed R version 4.1.2, specifically using the MatchIt package. This approach allowed us to effectively control for confounders and estimate the intervention’s impact more accurately. Statistical significance was set at *p* < 0.05 across all analyses.

## Results

### Study population characteristics

A total of 547 patients who were treated through MABS or COBS at our hospital between June 2020 and July 2023. After propensity score matching with ratio 1:1, 232 patients were considered for final analysis, including 116 in MABS-group and 116 in COBS-group (Fig. 1). Table 1 presents baseline characteristics before and after propensity score matching. Before matching, there were no significant differences between the groups in terms of BMI, pathological type, hormone-receptor status, Her2 status, or Ki-67 status. However, significant differences were observed in age, tumor size, cancer stage, and lymph node status. Post-matching, there were no significant differences in these variables.

**Table 1:** Baseline characteristics before and after PSM

Parameters	Before PSM			After PSM		
	MABS (*n* = 116)	COBS (*n* = 431)	*P*	MABS (*n* = 116)	COBS (*n* = 116)	*P*
Age, yr	48.67 ± 5.32	50.53 ± 6.68	0.029	48.67 ± 5.32	49.26 ± 6.16	0.523
BMI (kg/m^2^) tumor size (mm)	23.69 ± 3.36 26.15 ± 11.52	24.39 ± 3.75 29.86 ± 12.34	0.153 0.023	23.69 ± 3.36 26.15 ± 11.52	24.03 ± 3.55 27.12 ± 11.67	0.540 0.602
Cancer stage 0-I II III	31 (39.7) 40 (51.3) 7 (9.0)	49 (25.4) 105 (54.4) 39 (20.2)	0.017	31 (39.7) 40 (51.3) 7 (9.0)	29 (37.2) 38 (48.7) 11 (14.1)	0.604
Lymph node status (n, %) Positive Negative	23 (29.5) 55 (70.5)	87 (45.1) 106 (54.9)	0.018	23 (29.5) 55 (70.5)	26 (33.3) 52 (66.7)	0.605
Pathologic type (n, %) Ductal Others	70 (89.7) 8 (10.3)	168 (87.0) 25 (13.0)	0.539	70 (89.7) 8 (10.3)	68 (87.2) 10 (12.8)	0.616
Hormone status (n, %) Positive Negative	56 (71.8) 22 (28.2)	148 (76.7) 45 (23.3)	0.398	56 (71.8) 22 (28.2)	58 (74.4) 20 (25.6)	0.718
Her2 status (n, %) Positive Negative	23 (29.5) 55 (70.5)	49 (25.4) 144 (74.6)	0.489	23 (29.5) 55 (70.5)	21 (26.9) 57 (73.1)	0.722
Ki-67 status (n, %) < 15% ≥ 15%	15 (19.2) 63 (80.8)	31 (16.1) 162 (83.9)	0.529	15 (19.2) 63 (80.8)	17 (21.8) 61 (78.2)	0.692

Values shown are mean ± SD or n (%). PSM, propensity score matching; MABS, minimally access breast surgery; COBS, conventional open surgery; BMI: body mass index; Her2: human epidermal growth factor receptor-2

### T lymphocyte subsets

**Fig. 3:**
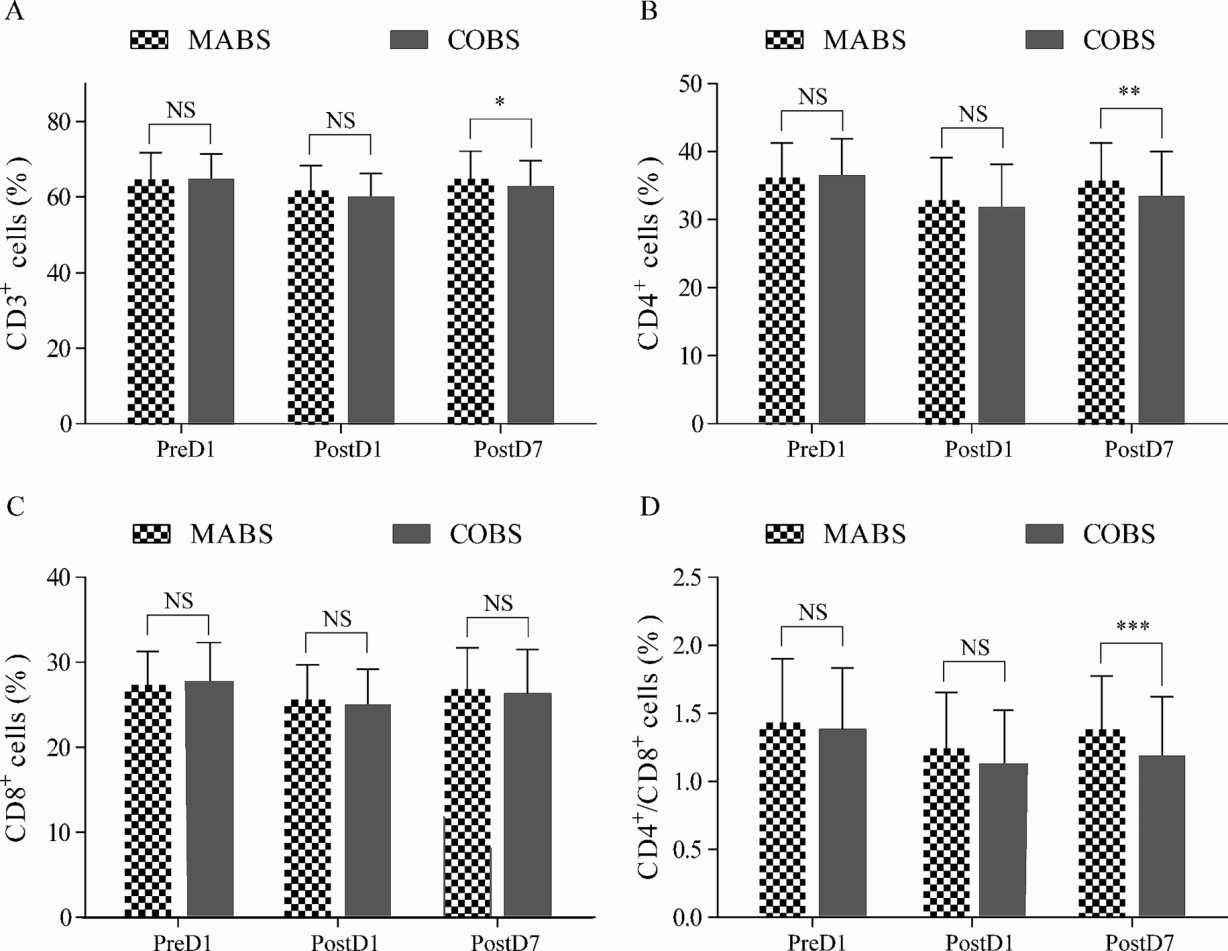
Comparison of proportions of (**A**) CD3^+^ cells, (**B**) CD4^+^ cells, (**C**) CD8^+^ cells, and (**D**) the CD4^+^/CD8^+^ ratio between MABS and COBS at various time points in patients. Values are expressed as mean ± SD. MABS, minimally access breast surgery; COBS, conventional open breast surgery; PreD, preoperative day; PostD, postoperative day; ns, no significance; *P < 0.05, **P < 0.01, ***P < 0.001

As shown in Table 2, both the MABS and COBS groups showed a significant decrease in the percentages of CD3+, CD4+, and the CD4^+^/CD8^+^ ratio from preoperative day 1 (PreD1) to postoperative day 1 (PostD1). In the MABS group, these parameters reverted back to preoperative levels by PostD7 (Fig. 2ABCD), while in the COBS group, they exhibited an increase from PostD1 to PostD7 but remained significantly lower than PreD1 (Fig. 2ABCD). The MABS group showed significantly higher percentages of CD3^+^, CD4^+^, and the CD4^+^/CD8^+^ ratio compared to the COBS group on PostD7 (Table 2, Fig. 3ABD), while CD8^+^ percentages exhibited similar trends between the two groups throughout all time points (Fig. 3C).

**Table 2:** Proportions of T lymphocyte subsets at different time points

Group and time point	Mean percent or ratio ± SD			
	CD3^+^	CD4^+^	CD8^+^	Ratio CD4^+^/CD8^+^
MABS (*n* = 116)				
PreD 1	64.88 ± 7.05	36.34 ± 5.11	27.37 ± 3.98	1.43 ± 0.47
PostD 1	62.05 ± 6.54^c^	33.03 ± 6.23^c^	25.66 ± 4.12^b^	1.24 ± 0.41^b^
PostD 7	65.12 ± 7.29^d^	35.86 ± 5.59^e^	26.92 ± 4.85	1.38 ± 0.39^f^
COBS (*n* = 116) PreD 1 PostD 1	65.05 ± 6.65 60.42 ± 6.17^c^	36.67 ± 5.36 32.03 ± 6.23^c^	27.83 ± 4.54 25.13 ± 4.12^c^	1.38 ± 0.45 1.14 ± 0.39^c^
PostD 7	63.12 ± 6.72^b^	33.63 ± 6.52^c^	26.36 ± 5.22^b^	1.19 ± 0.43^c^

*Abbreviations* PreD, preoperative day; PostD, postoperative day; MABS, minimally access breast surgery; COBS, conventional open breast surgery. ^a^*P* < 0.05, ^b^*P* < 0.01 or ^c^*P* < 0.001, PostD1 or PostD7 vs. PreD1 in same group; ^d^*P* < 0.05, ^e^*P* < 0.01, or ^f^*P* < 0.001, MOBS vs. COBS on PostD7.

### Intraoperative outcomes and postoperative complications

Surgical procedures were successful in both groups. MABS group had a higher average operating time (126.68 ± 18.59 min) compared to the COBS group (120.68 ± 17.32 min; *P* < 0.05). Additionally, the MABS group showed significantly lower levels of intraoperative blood loss, postoperative drainage, shorter extubation time, and hospital stays. Both groups had similar rates of postoperative complications (Table 3).

**Table 3:** Intraoperative outcomes and postoperative complications in patients

Parameter	MABS (*n* = 116)	COBS (*n* = 116)	*P*
Operating time, min	126.68 ± 18.59	120.68 ± 17.32	0.012
Retrieved lymph node Intraoperative blood loss, ml	16.35 ± 3.92 37.63 ± 5.13	15.15 ± 3.46 58.63 ± 8.68	0.014 < 0.001
Drain removal time, d	6.62 ± 1.53	8.82 ± 1. 92	< 0.001
Postoperative drainage volume, ml	182.75 ± 39.62	347.83 ± 62.39	< 0.001
Postoperative complications Infection Seroma or hematoma	2 6	3 7	0.651 0.775
Skin or nipple necrosis Flap loss or ischemia Fat necrosis	4 1 2	5 1 3	0.734 1.000 0.651
Postoperative hospital stay, d	11.12 ± 3.87	15.17 ± 4.54	< 0.001

Values shown are mean ± SD or n (%). MABS, minimally access breast surgery; COBS, conventional open breast surgery

## Discussion

In this study, we conducted a propensity-matched retrospective cohort analysis to compare postoperative immune function in MABS vs. COBS in treatment of breast cancer patients. The results suggest a decrease in postoperative immune function for both MABS and COBS. Notably, however, immune suppression was less severe and recovery quicker in patients who underwent MABS.

Before applying PSM, there were notable differences in age, tumor size, cancer stage, and lymph node status, all of which favored the COBS group. These disparities could potentially bias the comparison between the two surgical methods. However, after implementing the matching process, these significant differences were eliminated, indicating that the groups had comparable baseline characteristics. This crucial adjustment allows us to more confidently attribute any observed variations in postoperative immune function to the surgical method itself, rather than to underlying differences in patient demographics or disease severity. PSM neutralized key differences in clinicopathologic variables, ensuring similar baseline characteristics between the groups. This enhanced the reliability of our analysis and confirmed that our findings accurately reflect differences due to the surgical methods, not pre-existing disparities.

Lymphocytes, pivotal elements in the immune system, are responsible for both specific and nonspecific immune tasks crucial for tumor surveillance [28]. However, major surgery has been consistently associated with a decline in the quantity and functionality of T lymphocyte subsets, resulting in significant suppression of postoperative immune function [29]. Various factors, including anesthesia, intraoperative bleeding, tumor resection, and lymph node dissection, can influence the activation of T lymphocytes following surgery [30, 31]. As the body’s injury repair mechanism is initiated, immune function gradually recovers, and radical tumor resection can also contribute to the restoration of T lymphocyte function [32].

To investigate the impact of different surgical approaches on immune function, we compared peripheral blood T lymphocyte subsets between the MABS and COBS groups during the perioperative period. At PostD1, CD3^+^, CD4^+^, CD8^+^, and CD4^+^/CD8^+^ ratios were lower in both groups compared to their preoperative levels at PreD1, indicating short-term immune suppression in both groups. However, as postoperative recovery progressed, immune function improved. At PostD7, immune indexes significantly improved in both groups, but the MABS group recovered to preoperative levels (*P* > 0.05), whereas the COBS group did not fully recover (*P* < 0.01), suggesting a slower recovery of immune function after COBS surgery. Furthermore, at PostD7, CD3^+^, CD4^+^, and the CD4^+^/CD8^+^ ratios were significantly lower in the COBS group compared to the MABS group, suggesting more severe postoperative immune suppression in the COBS group. These results of this study demonstrates that MABS treatment preserves and restores preoperative immune function more effectively than COBS surgery, highlighting the potential advantages of MABS in enhancing patients’ immune responses.

Minimally invasive surgery, compared to open surgery, offers several advantages, including gentler tissue and organ traction, finer anatomical manipulation, reduced damage to blood vessels and lymphatic vessels, decreased trauma, fewer postoperative complications, and faster recovery [11, 33–36]. In our study, we observed that patients who underwent MABS experienced less bleeding, lower drainage volume, shorter extubation time, and shorter hospital stays compared to those who underwent COBS. These findings suggest that the favorable outcomes associated with minimally invasive surgery, such as reduced blood loss and trauma, may contribute to the less severe immune suppression observed in patients undergoing this approach.

Despite slower immune recovery in the COBS group, there was no significant rise in postoperative infection rates, which can be attributed to multiple factors. Firstly, not only immune status but also effective intraoperative and postoperative measures, including antibiotic use and good hygiene, likely helped prevent infections. Secondly, the expected direct link between immune function and infection risks may not be as strong, with individual health differences also impacting outcomes. Additionally, variations in how infections are diagnosed and recorded, as well as study limitations like sample size, could influence data interpretation. Therefore, despite delayed immune recovery, effective management and other variables ensured stable infection rates, suggesting the need for more research into how immune function affects infection risks post-surgery.

Our study has limitations that should be acknowledged. It was a retrospective study, which may introduce selection bias despite our efforts to minimize it. Further confirmation through multi-center, prospective, and larger sample studies is necessary. Additionally, our immune function assessment was limited to specific time points before and after surgery. Extending the observation period and exploring additional immune indicators would enhance our understanding. It is also important to directly examine cellular immune function in tumor specimens to validate our findings. These limitations emphasize the need for further research to strengthen and broaden our understanding in this field.

## Conclusions

Despite these limitations, our study provides evidence to support the potential of MABS in minimizing postoperative immune suppression. This highlights the necessity for larger randomized clinical trials and mechanistic studies to elucidate the immune preservative mechanisms of MABS.

## Data Availability

No datasets were generated or analysed during the current study.
